# Interspecific Introgression in Cetaceans: DNA Markers Reveal Post-F1 Status of a Pilot Whale

**DOI:** 10.1371/journal.pone.0069511

**Published:** 2013-08-19

**Authors:** Laura Miralles, Santiago Lens, Antonio Rodríguez-Folgar, Manuel Carrillo, Vidal Martín, Bjarni Mikkelsen, Eva Garcia-Vazquez

**Affiliations:** 1 Department of Functional Biology, University of Oviedo, Oviedo, Asturias, Spain; 2 Instituto Español de Oceanografía, Vigo, Galicia, Spain; 3 G.R.E.M.MAR Dolphin Rescue and Research Group of Marine Mammals, Cámpelo Parroquia de San Juan de Poio, Galicia, Spain; 4 Canarias Conservación Cetacean Research Society, La Laguna, Canary Islands, Spain; 5 Sociedad para el Estudio de los Cetáceos en el Archipiélago Canario (SECAC), Yaiza, Canary Islands, Spain; 6 Faroese Museum of Natural History, Tórshavn, Faroe Islands; Natural History Museum of Denmark, Denmark

## Abstract

Visual species identification of cetacean strandings is difficult, especially when dead specimens are degraded and/or species are morphologically similar. The two recognised pilot whale species (*Globicephala melas* and *Globicephala macrorhynchus*) are sympatric in the North Atlantic Ocean. These species are very similar in external appearance and their morphometric characteristics partially overlap; thus visual identification is not always reliable. Genetic species identification ensures correct identification of specimens. Here we have employed one mitochondrial (D-Loop region) and eight nuclear loci (microsatellites) as genetic markers to identify six stranded pilot whales found in Galicia (Northwest Spain), one of them of ambiguous phenotype. DNA analyses yielded positive amplification of all loci and enabled species identification. Nuclear microsatellite DNA genotypes revealed mixed ancestry for one individual, identified as a post-F1 interspecific hybrid employing two different Bayesian methods. From the mitochondrial sequence the maternal species was *Globicephala melas*. This is the first hybrid documented between *Globicephala melas* and *G. macrorhynchus*, and the first post-F1 hybrid genetically identified between cetaceans, revealing interspecific genetic introgression in marine mammals. We propose to add nuclear loci to genetic databases for cetacean species identification in order to detect hybrid individuals.

## Introduction

In a progressively threatened oceanic environment where large species are more and more endangered, cetacean monitoring is increasingly important for estimating population censuses and early detecting signals of species depletion [1]. However, visual species identification is not always accurate. Some species are morphologically similar and their distribution ranges overlap. A correct taxonomic identification is indeed important for practical issues of management and conservation of cetacean species.

The two recognised pilot whale species, *Globicephala melas* and *G. macrorhynchus*, are cetaceans of charismatic behaviour. They are highly social and exhibit post-reproductive female care. They are sympatric across a North Atlantic latitudinal area from American to European coasts [2], [3]. Based on their osteology, Van Bree [4] demonstrated that they are two clearly distinct species; however, their external appearance is similar and the morphometric characteristics employed to visual species discrimination partially overlap [5]. Therefore species identification based on external morphology may be difficult and in some cases impossible [6]. Moreover, dead stranded individuals are sometimes highly degraded and their distinctive traits may be lost. For these reasons, as in other forensic zoological studies, DNA-based identification is necessary [7], [8].

Genetic species identification in cetaceans is generally based on maternally inherited mitochondrial DNA (mtDNA). A cetacean sequence database, DNA Surveillance [8], has been created to help in this purpose. It contains reference sequences for all known cetacean species. In addition, other databases like for example GenBank [9] have also reference sequences for cetaceans –and many other organisms. Although mtDNA is more frequently used for cetacean identification [7], [8] nuclear markers may be also needed for this purpose. Some species naturally hybridize *e.g.* [10], [11], [12]. Hybrids may exhibit morphologically ambiguous phenotypes *e.g*. [13] and therefore nuclear (biparentally inherited) genetic markers are needed for accurate identification of pure species and their hybrids [14]. Nuclear markers are also recommended in cases of PCR contamination [14], *e.g.* bacterial contamination of cetacean tissues. In this study, we have used mitochondrial and nuclear genetic markers to determine the species of stranded individuals and to investigate the possible existence of some genetic mixture between the two pilot whale species found in waters off northern Spain.

## Materials and Methods

**Figure 1:**
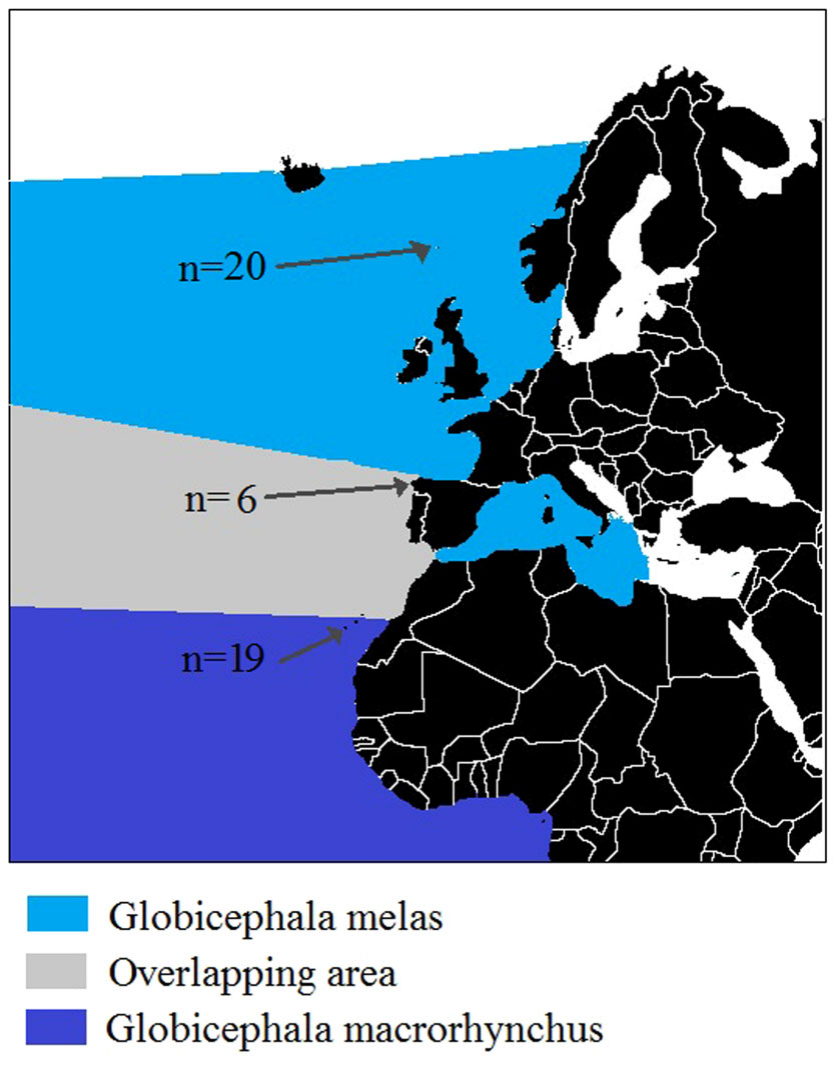
Pilot whale NE Atlantic distribution and sampling areas. Faroe Islands (n = 20), Galicia region (n = 6) and Canary Islands (n = 19).

Six stranded pilot whales (Table 1), 19 reference *Globicephala macrorhynchus* from Canary Islands and 20 reference *Globicephala melas* from Faroe Islands (donated by the Faroese Museum of Natural History) were analysed (Figure 1). All animal samples were obtained from dead specimens: dead strandings and museum collection. We obtained the CITES permit (ESBI00001/12I) and all the permissions from the Faroese Museum of Natural History to analyse the Faroese samples. No one animal suffered nor was injured or killed for this study. The protocol employed was approved by the Committee on the Ethics of Animal Experiments of the University of Oviedo.

**Table 1:** Stranded pilot whale samples analyzed.

	Reference	Visu	P	Sex	Size	Date	Location
Galicia01	69/85	*G. melas*	2	F	450	22/05/1996	42°34′42″N 09°05′07″W
Galicia02	72/77	*G. melas*	3	M	496	14/12/1995	42°16′39″N 08°29′50″W
Galicia03	74/84	*G. melas*	2	F	450	17/05/1996	42°53′33″N 09°15′51″W
Galicia04	75/103	*G. macrorhynchus*	3	F	391	9/09/1998	43°44′00″N 07°40′19″W
Galicia05	77/N	Not possible	4	M	553	20/03/2005	-
Galicia06	78/G	*G. melas*	2	F	360	8/12/2011	42°23.429N 08°49.894W

Visual identification done by experts in cetaceans. P: State of preservation proposed by the European Cetacean Society (ECS) ranged from 2 (freshly dead) to 4 (highly degraded); M: male; F: Female; -: unknown location (accidental capture by-catch).

DNA was extracted with a Chelex-based protocol [15]. The mitochondrial control region (D-loop) was amplified following Oremus *et al.* [16]. Sequences were edited with BioEdit Sequence Alignment Editor [17]. NCBI-BLAST [18] and DNA Surveillance [8] online software were employed for species identification. The number of haplotypes, haplotypic and nucleotidic diversities were calculated with DNAsp v5 [19]. A Neighbour-Joining tree with 1 000 bootstrap re-sampling was reconstructed from sequences with PHYLIP v.3.69 [20].

Eight microsatellite loci (EV37MN; EV94MN; 199/200; 415/416, 417/418, 409/470; 468/469 and 464/465) were amplified as in Fullard *et al.* [21] and genotyped employing GeneMapper® Software. A multi-tube method [22] was employed to validate the allele scores. Each microsatellite locus was individually amplified four times in three different thermal cycler machines (Applied Biosystems 2720 Thermal Cycler). Scoring errors, large allele dropout and null alleles were checked with MICROCHECKER [23]. Linkage disequilibrium tests were performed with GENEPOP version 4.2 [24]. Variation parameters (number of alleles; allele richness; minimum, maximum and mean allele length; expected and observed heterozygosities; F_IS_) and distances between genotypes for populations (Nei distances) and individuals (Fuzzy set similarity distances) were calculated with Microsatellite Analyser MSA 4.05 [25]. F_ST_ distances were calculated with Arlequin v.3.5.1.3 [26] with 1 023 permutations and 0.05 significance level. Neighbour-Joining trees based on genetic distances and bootstrap (1 000 bootstrapping) were reconstructed with PHYLIP v. 3.69 [20]. Species assignment was done using three different methods widely employed for microsatellites *e.g*. [27]. The likelihood-based Bayesian method of Rannala and Mountain [28] was performed with GeneClass2 [29] with 0.05 score threshold. Two fully Bayesian methods were also employed: one with the program STRUCTURE 2.3.1 [30] (under the “Admixture model” which assumes that individuals may have mixed ancestry; burn-in period of 100 000 steps followed by 1 000 000 Markov Chain Monte Carlo (MCMC) iterations and five runs for k = 2 -two species), and other with NewHybrids 1.0 [31] software (with 500 000 sweeps after a burn-in period of 100 000 MCMC iterations) that identifies first and second generations hybrids and backcrosses.

## Results and Discussion

The markers employed exhibited sufficient variation for discriminating between the two pilot whale species in reference samples. The mitochondrial D-loop haplotypes were clearly species-specific, as expected [12]. Intraspecific polymorphism was found for the two species, with seven and two haplotypes for *G. macrorhynchus* and *G. melas* respectively (Table 2). Eight microsatellite loci were assayed, from which two (EV94MN and 468/469) exhibited possible null alleles in our dataset (detected with MICROCHECKER) and were discarded from further analyses. For the six remaining microsatellite loci, null alleles and linkage disequilibrium were found to be non-significant, allowing their use for genetic assignment. Allelic frequencies of the six selected microsatellite loci were deposited in LabArchives, LLC (DOI: 10.6070/H43F4MHJ) as well as all genotypes (DOI: 10.6070/H4765C78). The number of alleles per locus ranged from 2 to10 (Table 2). No significant differences between expected and observed heterozygosities and low F_IS_ values were found (Table 2). Highly significant F_ST_-values between species (0.2957, P<0.00001) confirmed enough resolution for species discrimination.

**Table 2:** Mitochondrial and nuclear variability of reference samples.

DNA marker		*G. melas*	*G. macrorhynchus*	All
D-loop	AN	KC542368 KC542369	KC542370 to KC542376	KC542368 to KC542376
	Nh	2	7	9
	Hd	0.105	0.692	0.681
	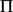	0.00018	0.00209	0.01243
EV37NM	A	6	7	8
	AR	5.916	6.640	7.168
	Ho	0.750	0.722	0.713
	He	0.700	0.722	0.740
	F_IS_	−0.046	0.029	
199/200	A	2	9	10
	AR	2.000	8.473	7.483
	Ho	0.150	0.333	0.365
	He	0.219	0.417	0.517
	F_IS_	0.337	0.286	
415/416	A	4	6	9
	AR	3.987	5.810	7.916
	Ho	0.800	0.667	0.767
	He	0.648	0.792	0.776
	F_IS_	−0.211	−0.039	
417/418	A	2	4	4
	AR	2.000	3.989	3.795
	Ho	0.350	0.400	0.466
	He	0.499	0.480	0.568
	F_IS_	0.321	−0.011	
409/470	A	7	10	11
	AR	6.678	10.000	8.819
	Ho	0.750	0.800	0.850
	He	0.789	0.560	0.761
	F_IS_	0.075	−0.173	
464/465	A	7	8	11
	AR	6.742	8.000	8.595
	Ho	0.789	0.667	0.752
	He	0.792	0.639	0.792
	F_IS_	0.031	0.067	
All loci	A	28	44	53
	Ho	0.598	0.769	0.652
	He	0.608	0.768	0.692
	F_IS_	0.041	0.109	0.029

For the mitochondrial control region: AN, accession numbers in the GenBank; Nh, number of haplotypes; Hd, Haplotype diversity; Π, Nucleotide diversity. For the microsatellite loci: A, number of alleles; AR, mean allelic richness; Ho and He, observed and expected heterozygosity respectively per locus and population; F_IS_, F_IS_-value per locus and population. P-values were not significant in any case.

**Figure 2:**
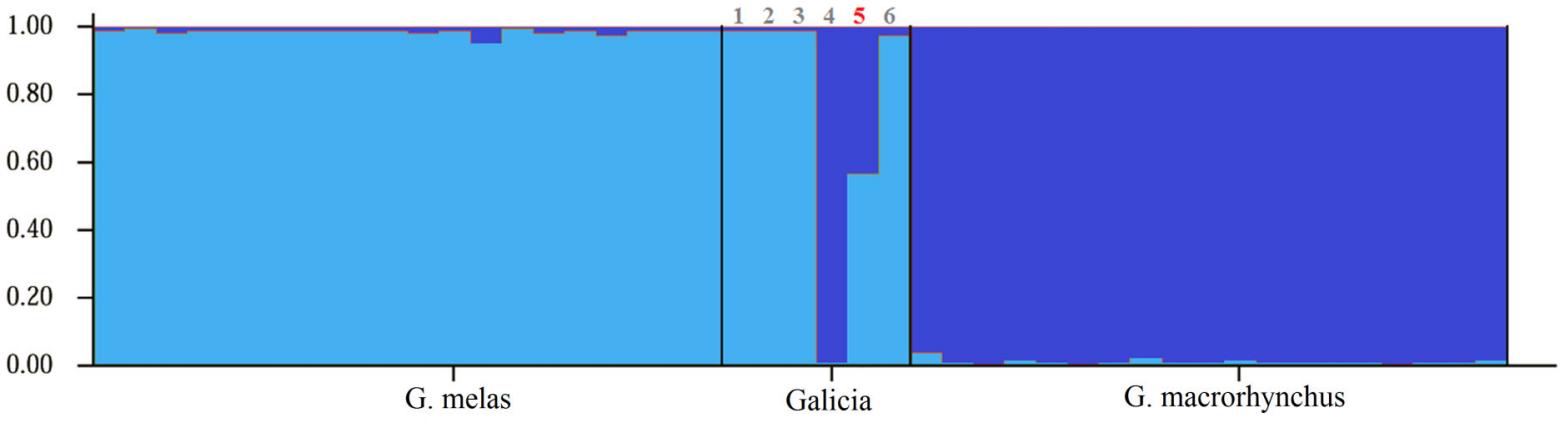
Individual membership of pilot whale samples from the considered regions estimated with STRUCTURE software. Each vertical bar represents one individual. Membership to *G. macrorhynchus* in dark blue and to *G. melas* in light blue. The numbers identifying stranded individuals are indicated above the corresponding vertical bars; the Post-F1 hybrid is in red.

**Figure 3:**
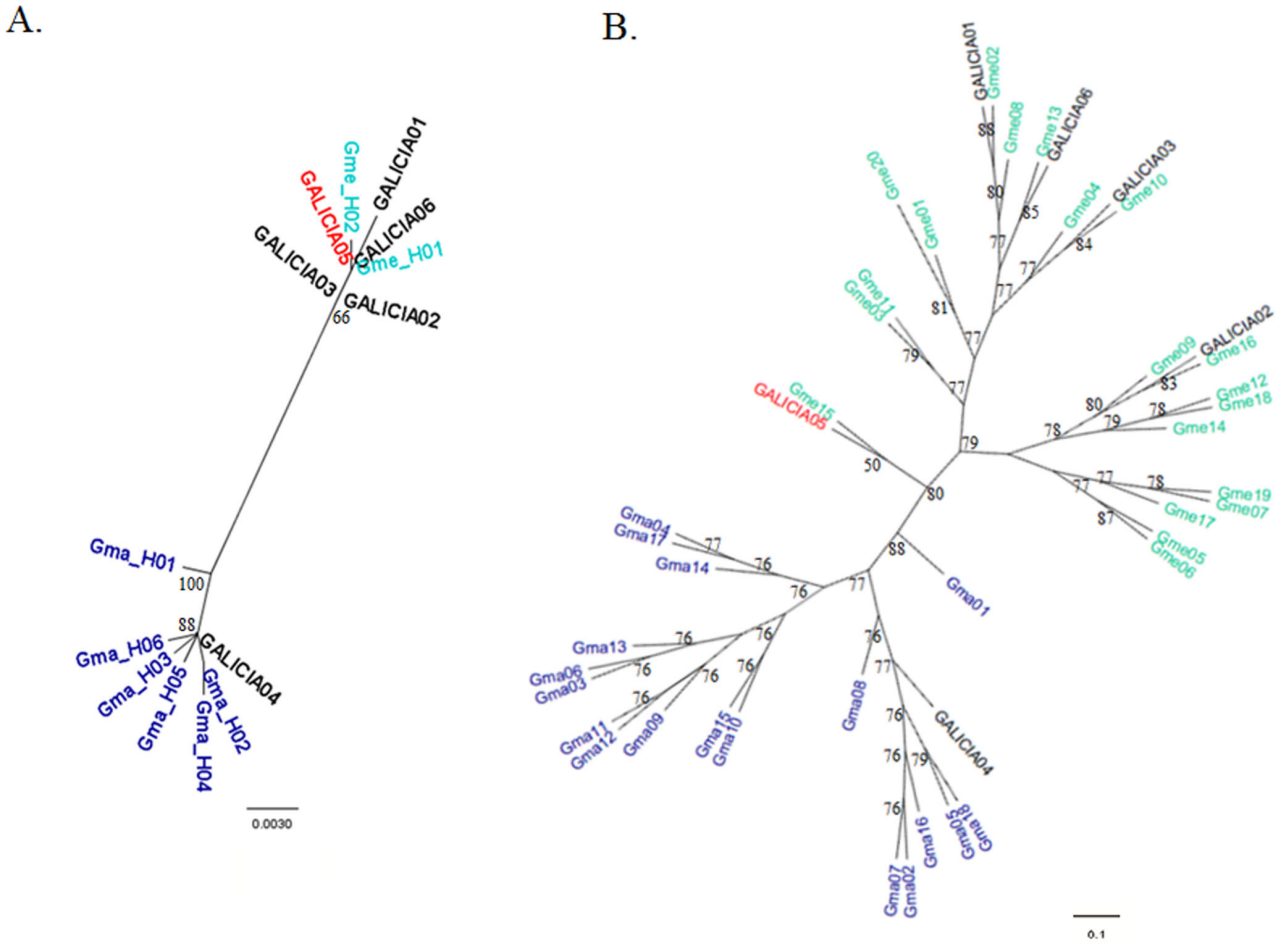
Mitochondrial and nuclear phylogenetic trees of the analyzed samples. Neighbour Joining trees reconstructed based on: A) mitochondrial D-Loop haplotypes; B) microsatellite loci genotypes. *G. macrorhynchus* is represented in dark blue and *G. melas* in light blue. Galician strandings are in black except the Post-F1 hybrid (Galicia05) that is in red. Bootstrapping is given for each node.

The six stranded pilot whale here analysed yielded positive amplification at the D-Loop sequence and the six microsatellite loci considered (Table 3); except for two loci that failed to amplify in one specimen (Galicia01). Genetic assignment was coincident with visual species identification when available (Table 4), and consistent for nuclear and mitochondrial markers. The male of ambiguous phenotype Galicia05 exhibited private alleles of the two parental species for 4 loci (Table 3 and Figures S1, S2, S3, S4, S5, S6): one exclusive allele of *G. melas* for EV37MN and 199/200, one exclusive allele of *G. macrorhynchus* at 464/465 locus and two alleles of *G. macrorhynchus* at 415/416 locus. These are unambiguous signals of post-F1 status. This individual was assigned with NewHybrids to a cross between F2 and *G. melas* (Table 4). The STRUCTURE software also revealed mixed ancestry for Galicia05 (57% membership of *G. melas*, 43% *G. macrorhynchus*; Figure 2). From the mitochondrial DNA its maternal species was *G. melas*. As in other studies [27], the two fully Bayesian methods (STRUCTURE and NewHybrids software) performed better than partially Bayesian assignment tests (GeneClass), which did not assign Galicia05 significantly to any species. The hybrid status of this individual is clearly visualized in the NJ tree reconstructed from nuclear markers (Figure 3): in the microsatellite-based tree, Galicia05 appears in the middle of the two species. Its clustering with a reference *G. melas* individual was not supported by bootstrapping, which was very low. In contrast the tree exhibited high bootstrapping in the rest of the nodes. These results therefore identify the first known hybrid between the two pilot whale species. These two species join the pairs blue whales and fin whales; Dall's and harbour porpoises; narwhals and belugas, and Risso's and bottle-nosed dolphins in the short list of sympatric cetaceans that hybridize [32]. A post-F1 hybrid foetus was described between blue and fin whales [13], but this is the first post-F1 adult cetacean documented until now and strongly suggests the possibility of interspecific introgression in marine mammals, a good example of Darwinian continuum between varieties and species [32].

**Table 3:** DNA markers of the stranded pilot whales analyzed.

	GenBank AN	Mitochondrial DNA	EV37MN	199/200	415/416	417/418	409/470	464/465
Galicia01	KC542377	*G. melas*	*184 , 184*	*114 , 114*	*236 , 236*	-	-	150 , 152
Galicia02	KC542368	*G. melas*	186 , 186	*114 , 114*	*234 , 236*	187 , 187	180 , 188	150 , 152
Galicia03	KC542378	*G. melas*	*184* , 196	*114* , *114*	*234 , 236*	187 , 187	180 , 188	150 , 150
Galicia04	KC542370	*G. macrorhynchus*	192 , 196	**126 , 142**	**228** , **232**	183 , 183	188 , 190	142 , 152
Galicia05	KC542368	*G. melas*	*184* , 186	*114* , 132	**226** , **232**	183 , 187	188 , 190	**146** , 150
Galicia06	KC542368	*G. melas*	*184* , 196	*114* , *114*	230 , 230	183 , 187	188 , 188	152 , 152

GenBank AN, accession number of the D-Loop sequence obtained for each whale, available at http://www.ncbi.nlm.nih.gov/genbank/ Exclusive alleles of *G. melas* and *G. macrorhynchus* are marked in italics and bold respectively. Results were confirmed with a multi-tube method to validate the allele scores. The suspected hybrid (Galicia05) has private alleles of both species.

**Table 4:** Species assignment of stranded pilot whales based on genetic markers.

	Mitochondrial DNA		Nuclear microsatellite loci		
	NCBI-BLAST	DNA-Surveillance	GC2	STRUCTURE	NewHybrids
Galicia01	*G. melas*	*G. melas*	*G. melas*	*G. melas* (0.99)	*G. melas*
Galicia02	*G. melas*	*G. melas*	*G. melas*	*G. melas* (0.99)	*G. melas*
Galicia03	*G. melas*	*G. melas*	*G. melas*	*G. melas* (0.99)	*G. melas*
Galicia04	*G. macrorhynchus*	*G. macrorhynchus*	*G. macrorhynchus*	*G. macrorhynchus* (0.98)	*G. macrorhynchus*
**Galicia05**	*G. melas*	*G. melas*	**Not significant**	***G. melas*** ** (0.57) ** ***G. macrorhynchus*** ** (0.43)**	**F2×** ***G. melas***
Galicia06	*G. melas*	*G. melas*	*G. melas*	*G. melas* (0.99)	*G. melas*

From mitochondrial D-Loop: online assignation with NCBI-BLAST [17] and DNA-Surveillance [8] software. From nuclear microsatellite loci: NewHybrids [28], GC2 GeneClass2 [26], STRUCTURE 2.3.1 [27] (membership to a species in parenthesis).

Finally, the present results emphasize the need of including nuclear markers in reference databases aimed at identifying cetacean species [8]. SNPs and nuclear sequence data, as well as hypervariable microsatellite loci, can be used for this purpose. As proposed long time ago by Palumbi and Cipriano [14], nuclear markers will help to understand the extent of interspecific hybridization in these marine mammals and its implications for conservation.

## Supporting Information

Figure S1 **EV37MN microsatellite chromatograms.** First graph *Globicephala melas*, second *Globicephala macrorhynchus*, and third sample Galicia 05.
(TIF)

Figure S2 **199/200 microsatellite chromatograms.** Graph order as in Figure S1.
(TIF)

Figure S3 **415/416 microsatellite chromatograms.** Graph order as in Figure S1.
(TIF)

Figure S4 **417/418 microsatellite chromatograms.** Graph order as in Figure S1.
(TIF)

Figure S5 **409/470 microsatellite chromatograms.** Graph order as in Figure S1.
(TIF)

Figure S6 **464/465 microsatellite chromatograms.** Graph order as in Figure S1.
(TIF)
