# Supplementary figures and images for: Interspecific Introgression in Cetaceans: DNA Markers Reveal Post-F1 Status of a Pilot Whale
Source: PLoS One. 2013 Aug 19;8(8):e69511. doi: 10.1371/journal.pone.0069511 (PMC3747178; doi:10.1371/journal.pone.0069511)

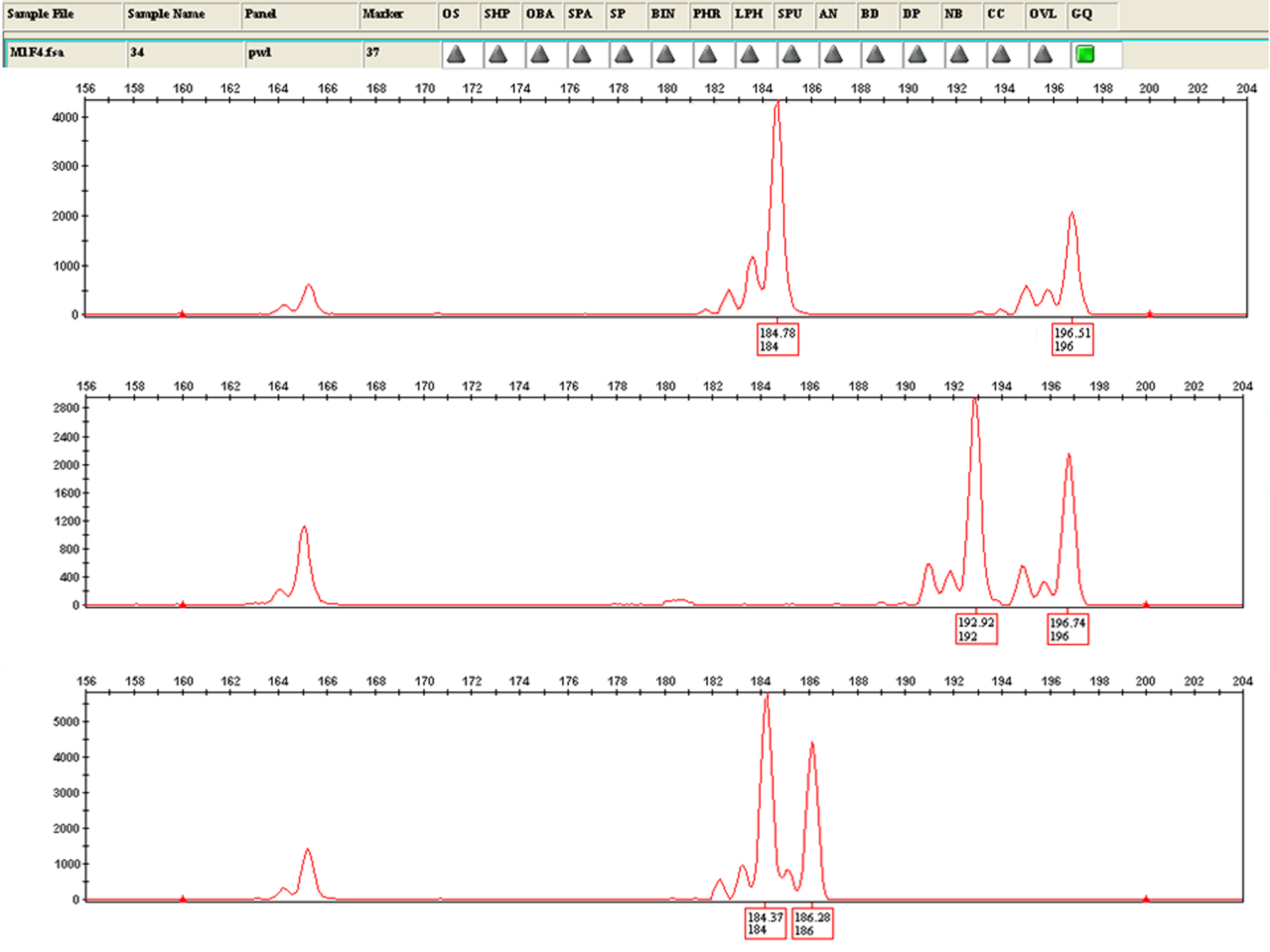

Supplement: Figure S1 — EV37MN microsatellite chromatograms. First graph Globicephala melas, second Globicephala macrorhynchus, and third sample Galicia 05. (TIF) [file pone.0069511.s001.tif]

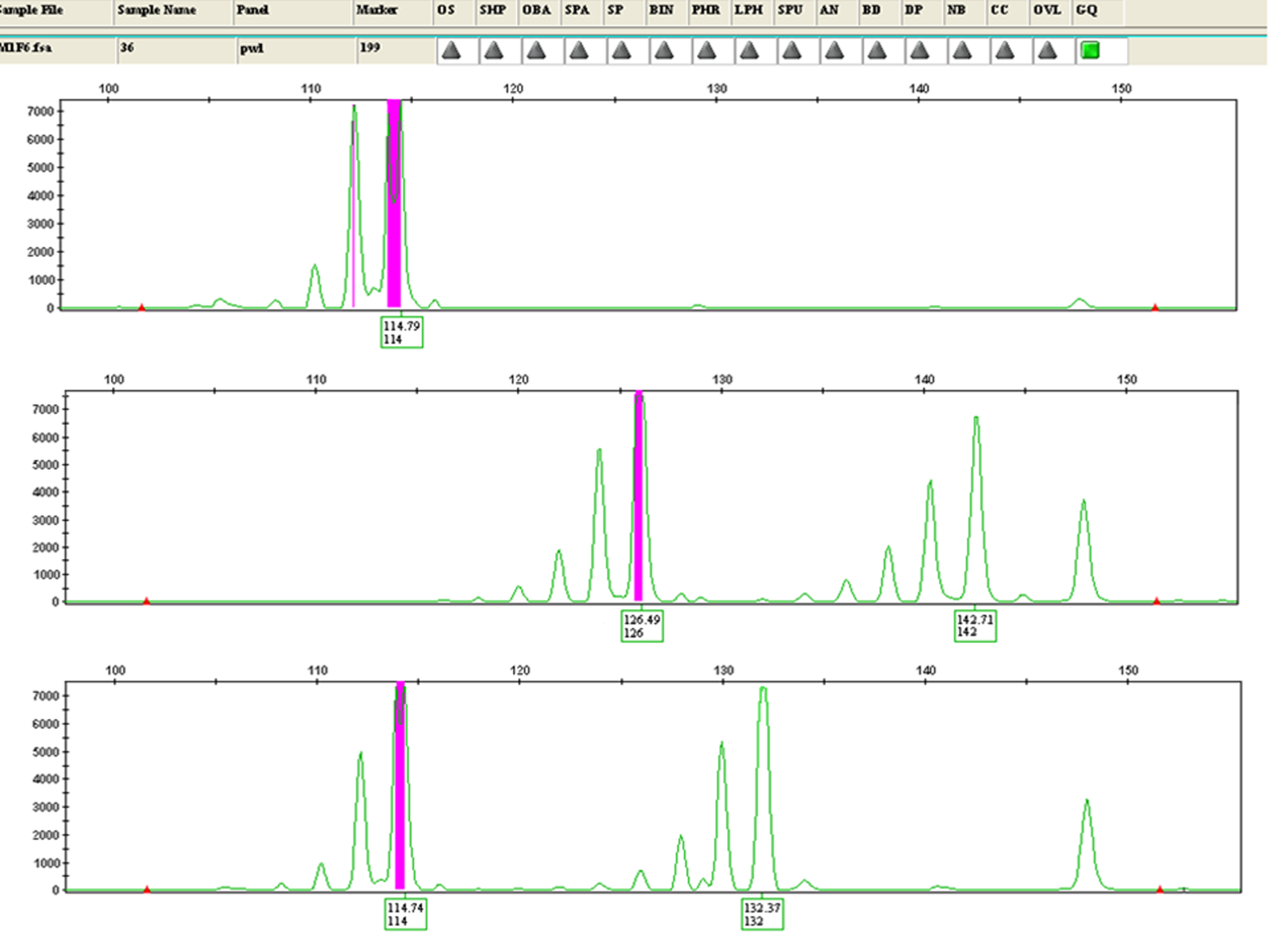

Supplement: Figure S2 — 199/200 microsatellite chromatograms. Graph order as in Figure S1. (TIF) [file pone.0069511.s002.tif]

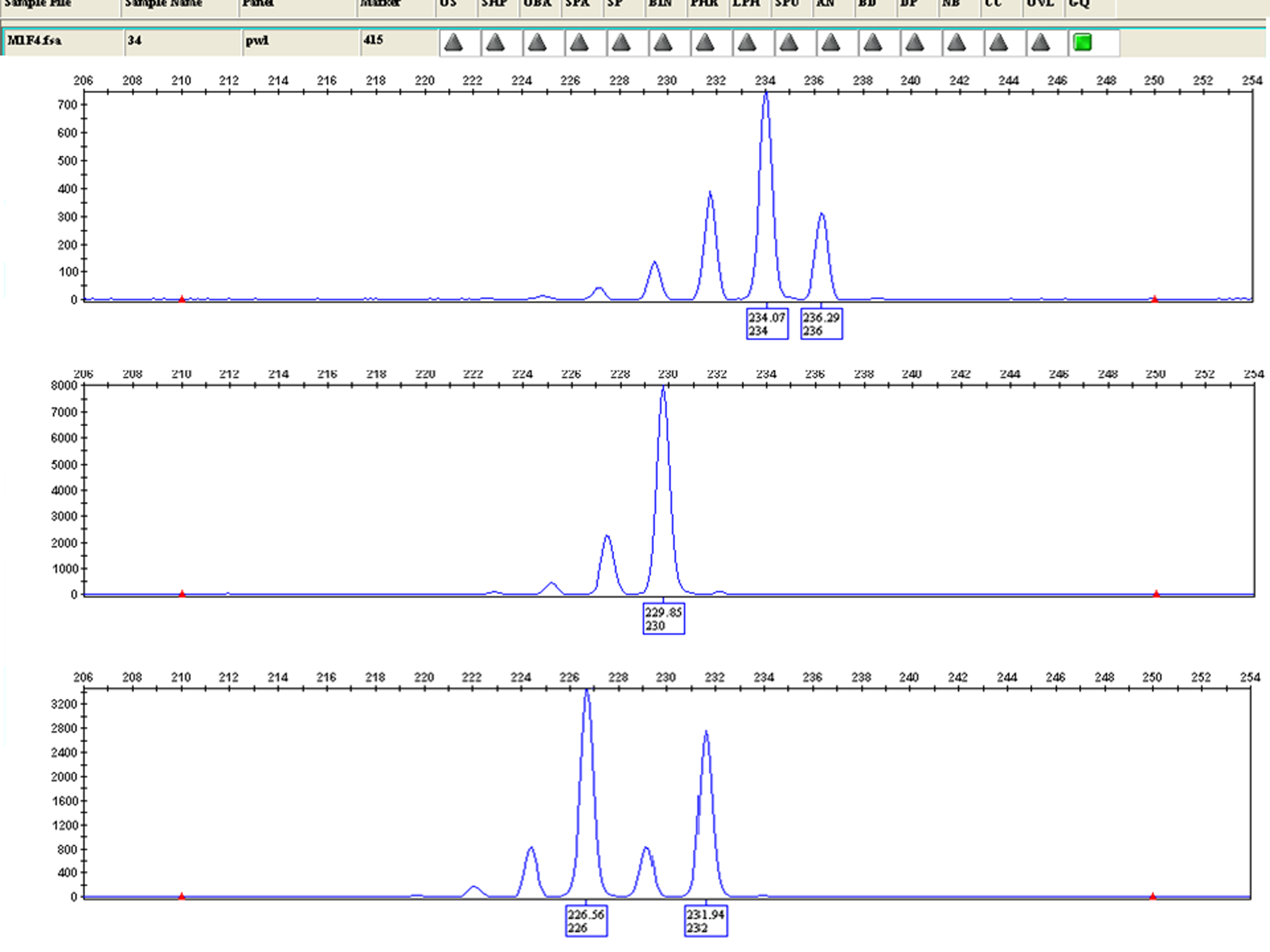

Supplement: Figure S3 — 415/416 microsatellite chromatograms. Graph order as in Figure S1. (TIF) [file pone.0069511.s003.tif]

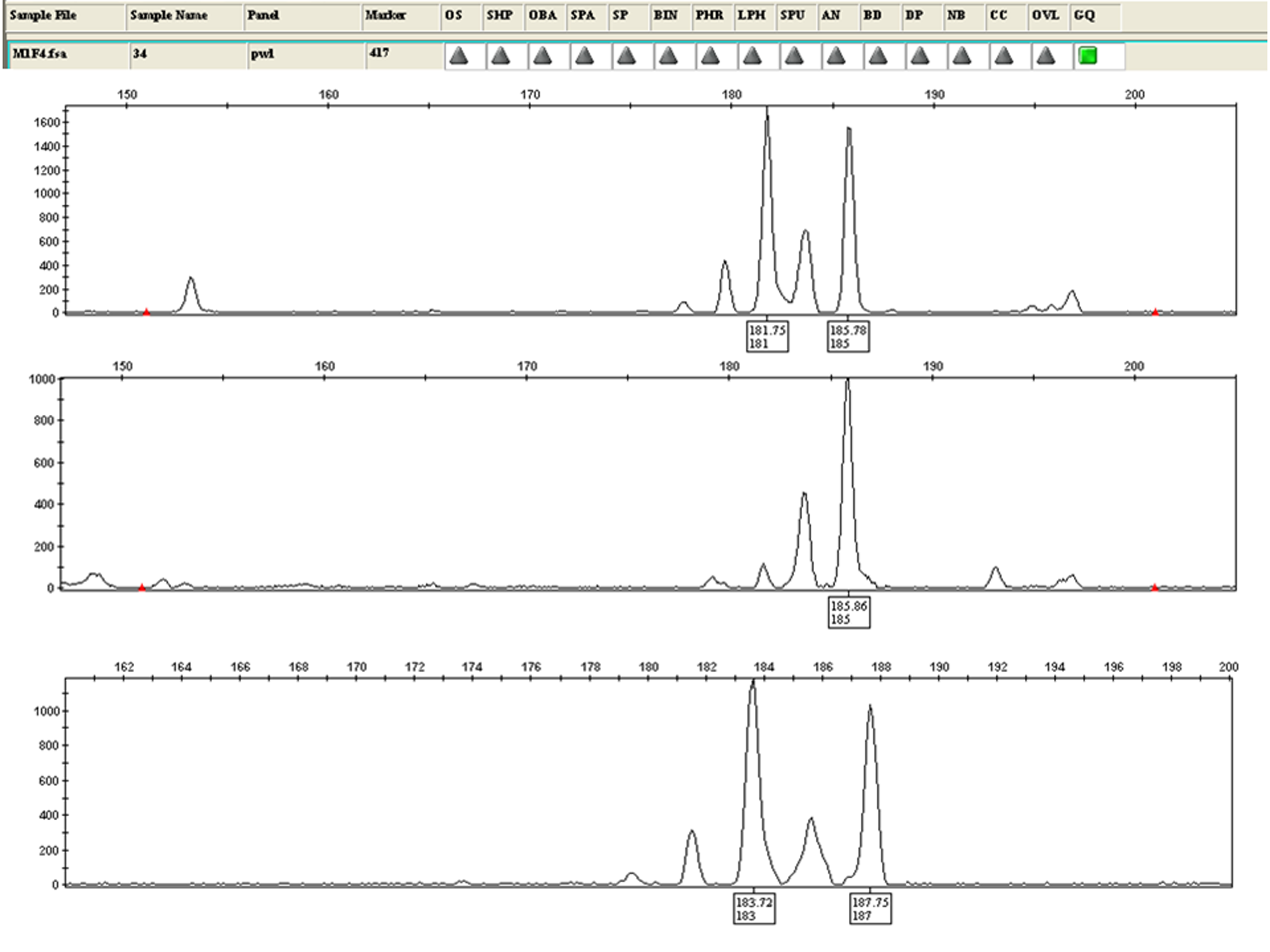

Supplement: Figure S4 — 417/418 microsatellite chromatograms. Graph order as in Figure S1. (TIF) [file pone.0069511.s004.tif]

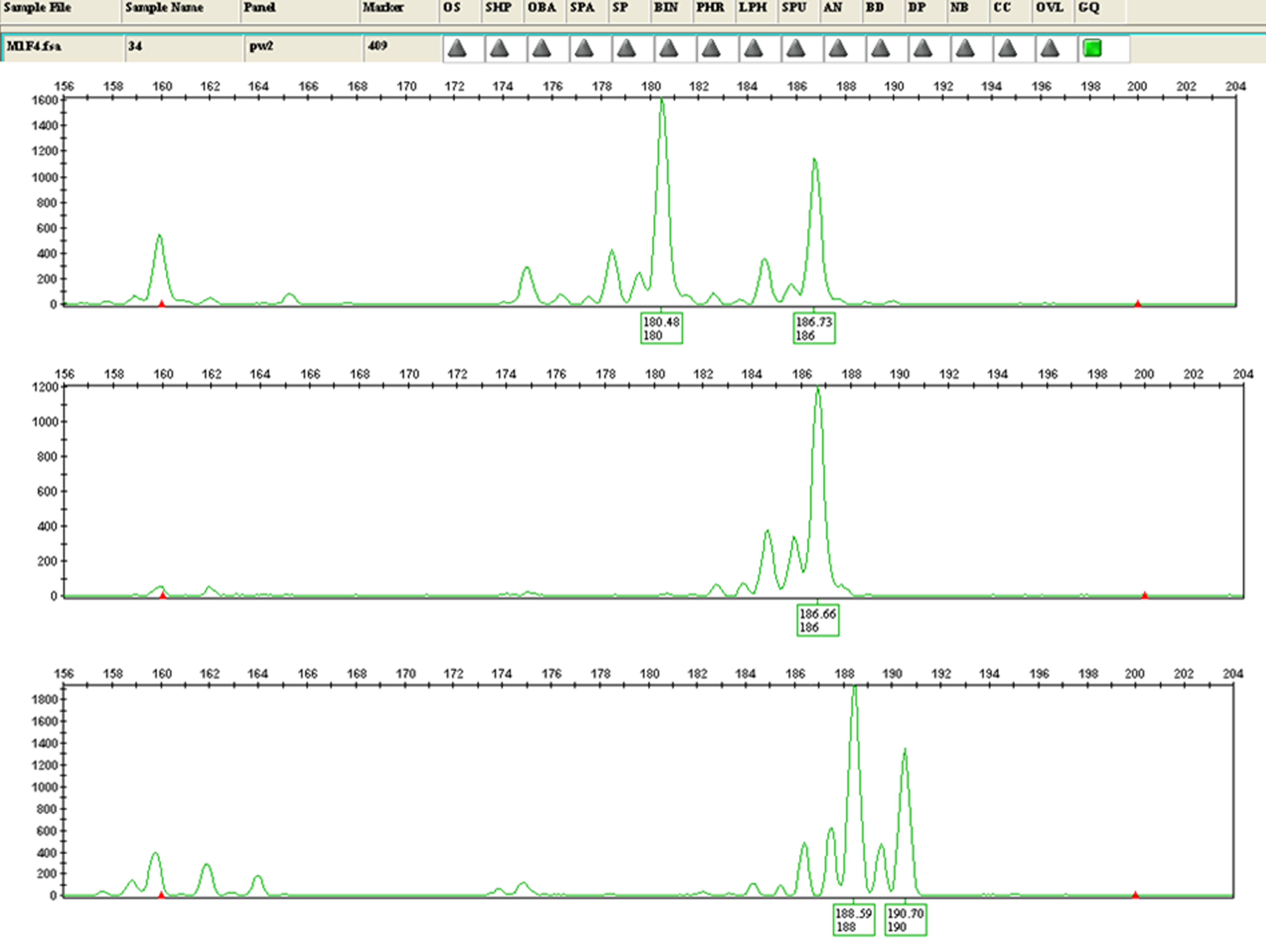

Supplement: Figure S5 — 409/470 microsatellite chromatograms. Graph order as in Figure S1. (TIF) [file pone.0069511.s005.tif]

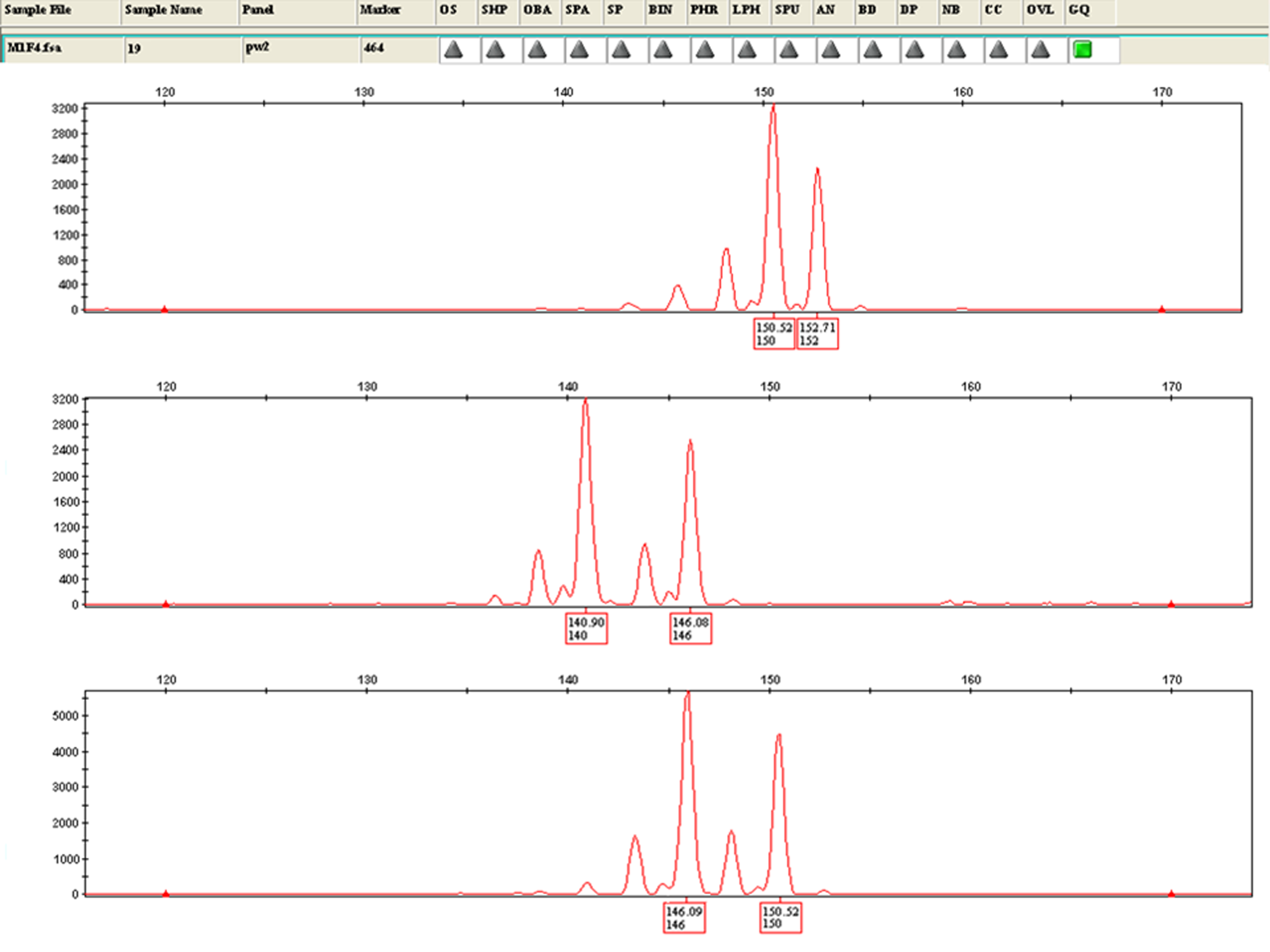

Supplement: Figure S6 — 464/465 microsatellite chromatograms. Graph order as in Figure S1. (TIF) [file pone.0069511.s006.tif]
